# Utility of the waist-to-height ratio, waist circumference and body mass index in the screening of metabolic syndrome in adult patients with type 1 diabetes mellitus

**DOI:** 10.1186/1758-5996-6-32

**Published:** 2014-03-04

**Authors:** Aldo Ferreira-Hermosillo, Claudia Ramírez-Rentería, Victoria Mendoza-Zubieta, Mario A Molina-Ayala

**Affiliations:** 1 Endocrinology Experimental Investigation Unit, Hospital de Especialidades Centro Médico Nacional Siglo XXI, Instituto Mexicano del Seguro Social (IMSS), Cuauhtémoc No. 330, Colonia Doctores, México City, DF, Mexico; 2 Endocrinology Department, Hospital de Especialidades Centro Médico Nacional Siglo XXI, Instituto Mexicano del Seguro Social (IMSS), Cuauhtémoc No. 330, Colonia Doctores, México City, DF, Mexico

**Keywords:** Metabolic syndrome, Type 1 diabetes, Waist circumference, Waist-to-height ratio

## Abstract

**Background:**

The incidence of macrovascular complications and morbidities associated to metabolic syndrome are increasing in patients with type 1 diabetes mellitus (T1DM). The combination of T1DM with features of insulin resistance similar to that of type 2 diabetes (T2DM), sometimes called “double diabetes”, has been associated with central obesity. Since the most methods to accurately detect body fat and insulin resistance are not readily available, we propose that certain indirect indexes for detecting obesity as waist-to-height ratio, waist circumference and body mass index, may be useful when screening for metabolic syndrome in patients with T1DM.

**Methods:**

We performed a transversal evaluation (clinical and biochemical) in all the patients of the T1DM Clinic (n = 120). We determined the presence of metabolic syndrome according to the Joint Statement Criteria by the American Heart Association/ National Heart Lung and Blood Institute and the International Diabetes Federation and the utility of certain anthropometric indexes for predicting double diabetes was evaluated.

**Results:**

Thirty seven percent of the patients were considered to have metabolic syndrome using these criteria (n = 30). These patients were significantly older (p = 0.002), have a higher glycated hemoglobin (p = 0.036), cholesterol (p < 0.012) and triglyceride concentration (p < 0.01) as well as body mass index (p = 0.004), waist circumference (p = 0.01) and waist-to-height ratio (p < 0.01) than the group without metabolic syndrome. Also their c-HDL is lower (p < 0.01). A value of 0.52 for waist-to-height ratio correctly classified the largest number of patients (68% of correctly classified) well as the waist circumference (66% of correctly classified) with an adequate specificity and sensibility. Meanwhile the most precise body mass index value only classified correctly to 61% of patients.

**Conclusion:**

Our data show that waist circumference and waist-to-height ratio indexes are useful to predict the presence of metabolic syndrome in adult patients with type 1 diabetes mellitus.

## Background

Type 1 diabetes mellitus (T1D) is an autoimmune disease in which pancreatic β cells are destroyed, generating an incapacity to maintain appropriate insulin and glucose concentrations [1]. The Instituto Mexicano del Seguro Social (IMSS) is a governmental institution that provides medical health for approximately 30% of the Mexican population. One of its recent publications reported a significant increase in the incidence of type 1 diabetes in the population evaluated by this healthcare system. Between the years 2000 and 2010, for the group under 19 years of age, the incidence increased from 3.4 to 6.2 per 100,00 in both sexes (p <0.001) [2].

Diabetes is one of the main causes of incapacity worldwide and affects all age groups. Years ago, microvascular complications, such as nephropathy, constituted the main cause of morbidity and mortality among patients with T1D [3]. More recently, there has been an increase in the macrovascular complication rate, associated with the presence of other cardiovascular risk factors such as hypertension, hypertriglyceridemia, hypoalphalipoproteinemia and central obesity, all of them in the context of metabolic syndrome (MS) [4]. Mexico has one of the largest prevalence in obesity worldwide, which has also affected the population with T1D [5]. Teupe and Bergis described the presence of pathologies commonly associated with type 2 diabetes in patients with T1D and named them “double diabetes” [6]. Even when there have been controversies regarding the use of this term and its diagnosis, these associations are being reported frequently. The treatment costs and complications will theoretically increase in the next years in patients fulfilling the criteria for MS. Accurate screening, early diagnosis and prevention will be important interventions for this group in the upcoming years.

The pathophysiology of MS is complex and it is yet being investigated. It has been hypothesized that the increased visceral adiposity in central obesity is a cardinal factor. Fat is a highly metabolic tissue that generates adipokines, greatly responsible for the development of insulin resistance [7]. Specific methods for visceral adiposity quantification such as axial tomography, magnetic resonance and electric bioimpedance are expensive and, for the most part, inaccessible in developing countries [8]. Therefore, surrogate measurements such as body mass index (BMI) and waist circumference (WC) have been used to assess the risk of MS [9]. All of them have serious limitations: race and clinical characteristics are two of the major determinants for the different results published by each author, making it necessary to validate every index for our specific population.

The waist-to-height ratio (WHtR) has proved to be a better discriminator for the metabolic and cardiovascular risk factors, however, studies differ in their cut-off values and usefulness to detect MS in general population [10]. To our knowledge, this information is not yet available for the population with T1D.

The purpose of this study was to evaluate and compare the utility of WHtR, BMI and WC in the detection of MS in patients with T1D and proposing specific cut-off values for the Mexican population.

## Methods

We performed a transversal evaluation in all the patients in the Type 1 Diabetes Clinic (Hospital de Especialidades Centro Médico Nacional Siglo XXI, tertiary referral center). We included 120 patients with the following characteristics: 18 years of age or older, at least 3 regular yearly visits to the clinic, no infections recorded in the 3 months previous to the study and no change in insulin dose during the same time. Patients with incomplete records or follow-ups, poor treatment adherence and primary dyslipidemias were excluded. The study completed all the requirements of the local ethics committee (Comite Local de Investigación y Ética en Investigación en Salud). The protocol and the aim of the study were fully explained to the subjects, who gave their written consent.

### Diagnostic criteria for MS

We used the joint statement criteria by the American Heart Association/National Heart Lung and Blood Institute (AHA/NHLBI) and the International Diabetes Federation (IDF) [11]. Patients were considered to have MS with 3 or more of the following components: serum triglycerides >150 mg/dl (1.7 mmol/l), serum high-density lipoproteins cholesterol (c-HDL) <40 mg/dl (1.03 mmol/l) in men and <50 mg/dL (1.29 mmol/l) in women or a previously treated dyslipidemia, arterial blood pressure >130/85 mmHg in two different determination or patients receiving treatment with antihypertensive drugs. WC >90 cm in men and >80 in women. Since patients were treated for type 1 diabetes, they all fulfilled the criteria of fasting plasma glucose >100 mg/dl (5.6 mmol/l) at least once.

### Anthropometric measurements

At the time of evaluation we registered weight (kilograms) and height (meters), as well as WC (centimeters). A single investigator, using the same calibrated scale with an integrated stadiometer, performed anthropometric measurements. WC was determined at the middle point between the inferior rim of the last costal arch and the superior rim of the anterosuperior iliac spine. BMI was calculated as the weight divided by height to the square. Blood pressure was determined in the left arm, after 10 minutes in a resting position, during a fasting state, without coffee or tobacco ingestion in the last week. The sphygmomanometer was calibrated and values were averaged after 2 different measurements with a 5-minute difference between them.

### Biochemical determinations

Laboratory results were obtained with a 6 mL sample in BD Vacutainer (BD Franklin Lakes, New Jersey USA), centrifuged at 3150 *x g* for 15 minutes and serum was then analyzed with a kit for glucose, cholesterol, c-HDL and triglycerides (COBAS 2010 Roche Diagnostics, Indianapolis USA) using photocolorimetry with spectrophotometer Roche Modular P800 (2010 Roche Diagnostics, Indianapolis USA). c-HDL samples were treated with enzymes modified with polyethileneglycol and dextrane sulphate, analyzed with the same photocolorimetric technique. Glycated hemoglobin (HbA1c) was evaluated by turbidimetric immunoanalysis (COBAS 2010 Roche Diagnostics, Indianapolis USA). c-LDL was calculated with Friedewald formula: c-LDL (mg/dl) = CT mg/dl – (c-HDL mg/dl + triglycerides mg/dl/5) if triglycerides were <400 mg/dl [12].

### Statistic analysis

Data were analyzed with STATA v.11. Kolmogorov-Smirnov test was used to determine normality. Results are expressed accordingly with means and standard deviations (SD) or medians and interquartile ranges (IQR). To establish associations between quantitative variables, Student t test or Mann–Whitney U test were used. Qualitative variables were associated with **χ**^2^ or Fisher tests. Receiver Operating Characteristic curves (ROC) for the different indexes and their combinations were used to identify the best cut of point with 95% confidence intervals. The areas under the curve (AUC) were compared with **χ**^2^. Additionally correlations where performed using Pearson test. A *p* < 0.05 was considered to be significant.

## Results

### Baseline characteristics

**Figure 1:**
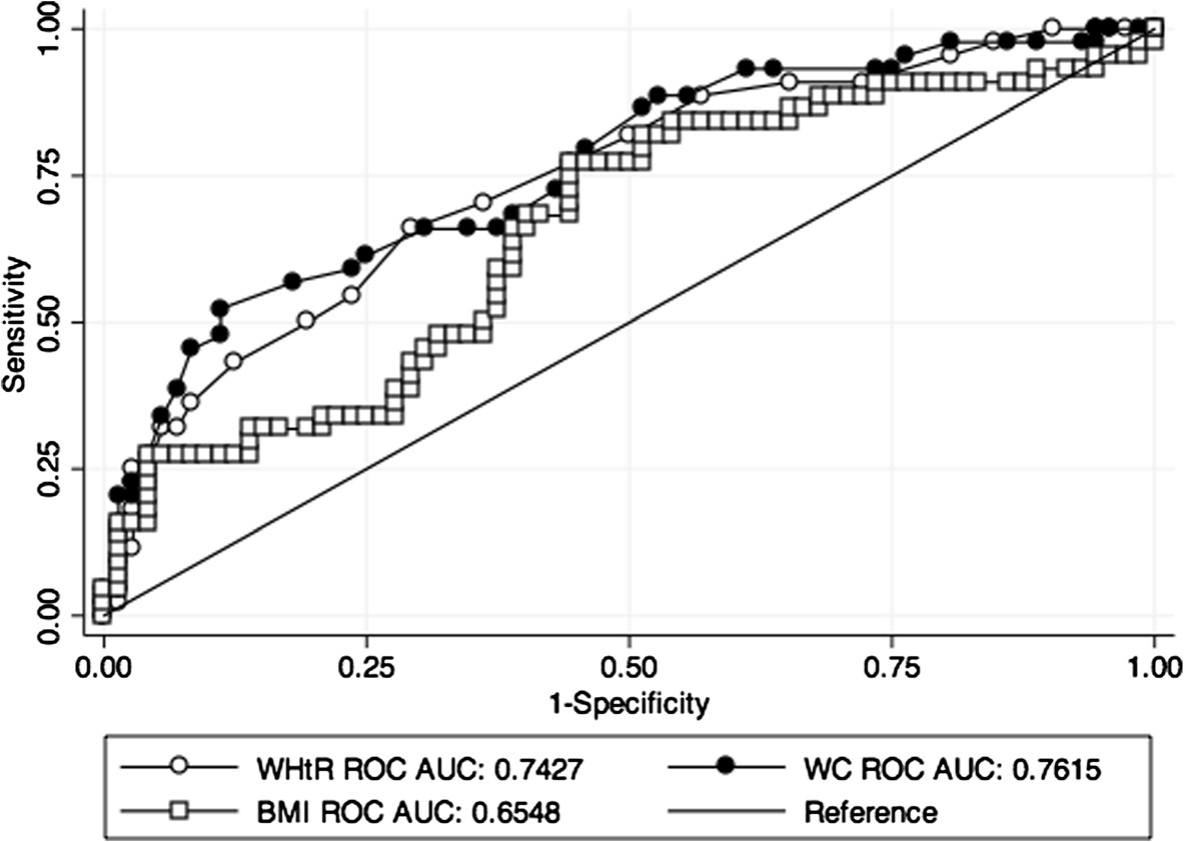
**ROC curves for WC, WHtR and BMI.** The ROC curves were performed to evaluate the best cut-off value for predicting metabolic syndrome in our population. WC = waist circumference, WHtR = waist-to-height ratio and BMI = body mass index.

Thirty seven percent of the patients were considered to have MS using these criteria (n = 30). Table 1 compares the baseline characteristics of the populations with and without MS. Patients with MS are significantly older, have higher BMI, WC and WHtR than the group without MS. Also, their HbA1c, cholesterol and triglyceride concentrations are higher while c-HDL is lower. This group had higher prevalence of hypertension and dyslipidemia.We performed a ROC curve for WHtR, BMI and WC in our population to determine the best cut off points to predict MS in our population (Figure 1).

**Table 1:** Baseline characteristics of the population comparing patients with metabolic syndrome (MS) and without MS

**Parameter**	**With MS**	**Without MS**	**p**
	**(n = 30)**	**(n = 90)**	
Age (years)	35 (26–42)	28 (20–34)	0.002*
Evolution of T1D (years)	20 (14.7–28)	16 (11–25)	0.051
Gender			
Female	26%	74%	0.584
Male	12%	88%	
Smoking status	5%	18%	0.551
Insulin dose (U/day)	49 (40–69.8)	45.5 (39–57)	0.086*
(U/kg/day)	0.69 (0.52–0.98)	0.75 (0.59–0.97)	0.974
Weight (m)	1.62 (1.57–1.70)	1.66 (1.55–1.67)	0.269
BMI (kg/m^2^)	25 (23.8–28.7)	23.3 (21.3–26.4)	0.004*
WC (cm)	90.4 ± 10.7	79.6 ± 8.2	<0.01^†^
Male	92.1 ± 12.6	85.2 ± 6.1	0.032
Female	89.6 ± 10.3	77.2 ± 7.88	<0.01
WHtR	0.54 (0.51–0.60)	0.49 (0.46–0.53)	<0.001*
Hypertension	14%	8%	<0.01^‡^
Dyslipidemia	18%	16%	0.02^‡^
Glycated hemoglobin (%)	8.8 (8–10)	8.2 (7.5–9.3)	0.036*
Total colesterol (mg/dl)	188 (169–210)	174.5 (153–206)	<0.012*
Tryglycerides (mg/dl)	175 (136–248)	88 (66–113)	<0.01*
c-HDL (mg/dl)	46 (37–53)	57 (48–66)	<0.01*
Male	41 (37–48)	49 (44–60)	0.044
Female	47 (37–55)	60 (53–69)	<0.01

Results are reported as Mean ± SD or mean and IQR.

*Mann–Whitney U test.

^†^Student t test.

^‡^X^2^ test.

Table 2 depicts the best cut off values, sensibility, specificity, likelihood ratio positive (LR+) and negative (LR-) predictive values and the percentage of correctly classified patients with each index. Likelihood ratios were used for assessing the value of performing these tests. A LR + higher than 1 indicates the test could be used to confirm the disease while a lower LR- indicates the test could be used to rule out the disease. The cut-off points were selected using Youden index, at which (sensitivity + specificity −1) is maximized [13]. A value of 0.52 for WHtR correctly classifies the largest number of patients with an adequate specificity and sensibility. This cut off was also appropriate for both sexes. When stratified, the male sub group had an AUC of 0.67 (0.48 – 0.85), sensibility of 62%, specificity of 61% and also 61% of correctly classified patients. In female patients the AUC was 0.79 (0.69 – 0.88), sensibility was 74%, specificity 68% and 70% of patients classified correctly (complementary data not shown). According to our results the WC cutoff value of 85 cm has lower sensitivity, specificity and detects fewer patients with MS compared to WHtR. Nevertheless, using cutoff values of 90 cm for men and 80 cm for women as recommended by IDF, increases the number of patients correctly classified to 76 and 73%, respectively.

**Table 2:** Best cutoff values to predict metabolic syndrome

**Index**	**AUC (IC 95%)**	**Cut off value**	**Sensibility**	**Specificity**	**% of correctly classified patients**	**LR+**	**LR-**
WHtR	0.75 (0.66–0.84)	0.52	70%	66%	68%	2.05	0.44
WC	0.76 (0.67–0.85)	85 cm	66%	65%	66%	1.89	0.52
Men	0.71 (0.49–0.93)	90 cm	69%	81%	76%	3.63	0.38
Women	0.80 (0.70–0.90)	80 cm	90%	63%	73%	2.42	0.15
BMI	0.66 (0.56–0.76)	24 kg/m^2^	78%	58%	61%	1.81	0.39

LR+: positive likelihood ratio. LR-: negative likelihood ratio. WHtR: waist-to-height ratio. WC: waist circumference. BMI: body mass index.

Considering that the patients with MS are older than patients without MS, we performed a Pearson correlation test. This test showed a small but positive correlation between age and WC (ρ=0.217; p = 0.017) but there was no correlation with WHtR index (p = 0.063). Nevertheless, there was a positive correlation in patients under 30 years of age (ρ=0.261, p = 0.039) which is not observed in older patients (p = 0.773). We didn’t observe any direct association between gender and MS even after dividing by age groups. Nevertheless if WC cutoff points are adjusted to gender, it reaches a MS detection of 76% for male and 73% for female.

Regarding the predictive power of BMI to detect MS, we found that the traditional cutoff value used to predict excess weight (BMI >25 kg/m^2^) had a sensibility of only 57% a specificity of 64% and a precision of 61%, while the cut off for obesity (BMI >30 kg/m^2^) had a lower sensibility (13%) but a high specificity (98%) with a precision of 67%.

These indexes may be proposed as one more of the screening tools easily available for clinicians in all centers. However, no single index seems to provide by itself the combination of high sensibility and specificity required for most diagnostic tests. However, the combination of these indexes may be helpful. For instance, combining the WHtR cutoff point of 0.52 with the BMI cutoff point of 24 kg/m^2^ presented a sensibility of 59%, but increased specificity to 83%, LR(+) 3.45, LR(−) 0.5 and a precision of 77%. When we analyzed the commonly used WHtR cutoff point of 0.5 and the cutoff of value for BMI of 25 kg/m^2^ we observed a lower sensibility of 52% with a similar high specificity of 81%, LR(+) 2.72, LR(−) 0.59 and a precision of 74%.

On the contrary, combining the WHtR cutoff point of 0.5 with the BMI cutoff point for obesity (30 kg/m^2^), increases sensibility to 86% with a lower, but useful specificity of 66%, LR(+) 2.55, LR(−) 0.22, however precision is only 14%.

Finally, using the χ^2^ test to evaluate the differences between the AUC, we found that when we used BMI as a reference parameter, both WHtR and WC predicted MS better (AUC BMI 0.65 *vs* AUC WHtR 0.74, p = 0.028 and AUC WC 0.76, p = 0.017). We didn’t find any statistically significant difference between WHtR and WC (p = 0.44).

## Discussion

Our study included a total of 120 adult patients with T1D (>18 years old) where we found a shocking 37% of patients fulfilling current criteria for MS using current diagnostic tools. Eighteen percent had dyslipidemia and 14% were hypertensive.

Recent data show that diagnosing and treating MS should be new concern for clinicians treating T1DM populations, considering the possibility of higher cardiovascular risk associated with it; however, the stratification is currently based on laboratory data, after the biochemical alterations are present [14]. To date, there is no single clinical, inexpensive, easily available index to predict the presence of MS in T1DM patients. Clinical data may help clinicians distinguish high-risk patients that may be candidates for additional interventions and studies. Primary prevention and low costs are important concerns for these decisions, especially in developing countries. Simple, but validated anthropometric measurements may be useful tools for these ends [15].

Our results are different from our previously reported data where the prevalence was 25% using the National Cholesterol Education Program: Adult Treatment Panel III (NCEP:ATPIII) criteria [16] and with the results of Thorn, *et al.* in the Finnish population (38% men, 40% women) [17]. We should consider the difficulties and controversies defining MS in T1DM patients and the limitations of using only clinical and general laboratory data in our study. Since all the patients had elevated serum glucose at least once, and this parameter is included as a diagnostic criterion, it could overestimate the prevalence of MS but if it is not considered, MS could be underestimated. However the same limitation will affect other publications if no other accurate system is proposed for the specific T1DM population.

Determining insulin resistance may also prove difficult in these patients. The gold standard to assess insulin resistance is the hyperinsulinemic euglycemic clamp. [18] However, this procedure is complex, expensive and it is usually not performed as part of the everyday evaluation of patients. It has been proposed that insulin dose could discriminate patients with insulin resistance; however, this is unreliable because it is inaccurately reported by patients and the potential effect of residual endogenous insulin secretion [19]. Nowadays, eGDR (estimated glucose disposal rate) index has been proposed for determining insulin resistance in patients with T1D. This study appears to adequately correlate with the clamp technique. [20] At this time, studies are being carried out to calculate specific cutoff points for this index in the Mexican population.

Clearly, MS in T1DM was a fairly unknown phenomenon until recent years. Along with the increasing incidence in obesity, T1DM patients are developing more features previously associated only with T2DM and may have higher cardiovascular risk in the future. A study in the Pittsburgh Epidemiology of Diabetes Complications (EDC) cohort studied 589 patients and showed a change in the BMI of T1DM patients in the last 20 years [21]. They found that obesity (BMI >30 kg/m^2^) rose up to 22.7% and overweight population (BMI 25–30 kg/m^2^) increased from 26 to a staggering 42% [22]. In the Mexican population, this increased weight has been attributed to two different but related factors: an exposure to calorie-rich diets and the over-insulinization associated with the intensive control therapies for T1D [23].

We don’t know yet the exact mechanisms by which MS increases the risk of cardiovascular events. Obesity may be a central factor in the pathophysiology of “double diabetes”. Some theories propose the participation of inflammatory cytokines secreted by the adipose tissue, known to generate atherothrombosis in type 2 diabetes [24-26]. Other studies have associated the increasing insulin concentrations to the stimulation of 11 β- hydroxysteroid dehydrogenase, which modifies the differentiation from fibroblasts to adipocytes and transformation from cortisone to the highly active cortisol [27].

Clinical data is important to detect risk factors for cardiovascular disease. BMI is one of the most frequently used indexes to detect obesity, however it can’t discriminate between excess adiposity and muscle mass [28] and it does not detect abdominal obesity. In our study we found that the traditionally used cut-off values to detect obesity had low sensibilities for MS. Another index, the WC correlates better with the abdominal adipose tissue and has been successfully associated with cardiovascular risk by different authors [29]. This index, however, may underestimate the real cardiovascular risk in patients with small stature, which may be important in many groups, such as ours [30].

It is still controversial which of the tree indexes is better predicting the development of MS. A Chinese study found that these three markers were useful to detect MS [31]. In the San Antonio Heart Study, BMI and WC were found to be equally useful in the Mexican-american population [32]. The INTERHEART study [33] and Bener, *et al.*[34] found that WC was the best index, while Ashweel, *et al.* found in 31 studies that WHtR was the best predictor [35]. It would seem that different ethnic groups require different indexes and that cut-off values must be standardized for each one of them.

Regarding the Mexican population, Elizondo-Montemayor, *et al.* found that children aged 6–12 years with the diagnosis of overweight and obesity had a 23% of MS with a cut off value of 0.59 LR(+) 3.80, LR(−) 0.232, sensibility of 81.8% and specificity of 78.5%, in contrast with the value of 0.50 found in the literature, which is supposed to have a sensibility of 100%, with a specificity of only 22.7%, LR(+) 1.29 and LR(−) 0.000 [36]. The same study showed that the 90^th^ percentile of WC had a precision of 82.6%, while the 97^th^ percentile of BMI had a precision of 76%.

However, none of these results reflects the adult population with T1D, which tends to be more obese. Our results show that there is a correlation between some of these indexes and age, but only in patients under 30 years of age. This may be due to the fact that all older patients tend to gain weight whether they develop MS or not. This may also mean that it could be more difficult to detect MS in T1DM patients as age increases and it confirms the need for preventive interventions at young ages.

In the current study, we found WC and WHtR to be the best predictive indexes for MS with a cut-off value of 0.52 (precision: 68%). Also, the proposed values for WC were highly sensitive and specific.

The preference of sensitivity *vs* specificity depends on the objective of the diagnostic tool. Most screening tools are selected when they are highly sensitive because of the reduced chance of false negative results and they are very useful when screening open populations, however higher specificities are required when we want to determine which patients should be referred for further evaluation given the high probabilities of MS. The final choice of one over the other may be in the hands of the clinician when a specific case presents before him.

On the other hand, for patients with a BMI over 24 kg/m^2^, the precision is high and using this index as a gold standard WC and WHtR are better markers for MS. This BMI cutoff is lower than the one proposed internationally to diagnose the overweight population, which suggests that our group may require a different set of diagnostic indexes in order to predict their cardiovascular risk.

This study is the first one to describe the utility of these indexes in adult patients with T1D. Using a clinical index is not a substitute for laboratory and specialized workup, however, when properly validated, may be a useful tool for clinicians worldwide. We propose that the combination of these simple indexes may be helpful for this and other larger populations similar to ours. Prospective studies with larger cohorts may be necessary to determine if it is applicable in other ethnic groups, their specific cut off points and the differentiation between sexes and age groups.

## Conclusion

WC and WHtR indexes are useful to predict the presence of metabolic syndrome in adult patients with T1D. We found that the WHtR index and WC allow for discrimination of patients with T1D and an increased probability of metabolic syndrome. The cut-off value of WHtR of 0.52 was the most accurate (68%) to discriminate MS in Mexican T1D. In ethnic groups where height variations could interfere with the precision of other indexes, WHtR could be a simple and useful tool to screen for cardiovascular risk factors in type 1 diabetes population.

## Abbreviations

AHA/NHLBI: American Heart Association/National Heart Lung and Blood Institute; AUC: Area under curve; BMI: Body mass index; c-HDL: Cholesterol associated to high density lipoproteins; c-LDL: Cholesterol associated to low density lipoproteins; HbA1c: Glycated hemoglobin a1c; IDF: International Diabetes Federation; IMSS: Instituto Mexicano del Seguro Social, Mexican Institute of Social Security; IQR: Interquartile ranges; LR: Likelihood ratio; MS: Metabolic syndrome; NCEP:ATPIII: National Cholesterol Education Program: Adult Treatment Panel III; SD: Standard deviations; T1D: Type 1 diabetes mellitus; WC: Waist circumference; WtHR: Waist-to-height ratio.

## Competing interests

The authors declare that they have no competing interests.

## Authors’ contributions

AFH and MAMA participated in the collection and analysis of data and in the writing of the manuscript. CRR and VMZ participated in the analysis of data and in the writing of the manuscript. All authors have read and approved the final version of the manuscript.
